# Factors associated with COVID-19 pandemic induced post-traumatic stress symptoms among adults living with and without HIV in Nigeria: a cross-sectional study

**DOI:** 10.1186/s12888-021-03617-0

**Published:** 2022-01-21

**Authors:** Morenike Oluwatoyin Folayan, Olanrewaju Ibigbami, Maha ElTantawi, Giuliana Florencia Abeldaño, Eshrat Ara, Martin Amogre Ayanore, Passent Ellakany, Balgis Gaffar, Nuraldeen Maher Al-Khanati, Ifeoma Idigbe, Anthonia Omotola Ishabiyi, Mohammed Jafer, Abeedah Tu-Allah Khan, Zumama Khalid, Folake Barakat Lawal, Joanne Lusher, Ntombifuthi P. Nzimande, Bamidele Emmanuel Osamika, Bamidele Olubukola Popoola, Mir Faeq Ali Quadri, Mark Roque, Anas Shamala, Ala’a B. Al-Tammemi, Muhammad Abrar Yousaf, Jorma I. Virtanen, Roberto Ariel Abeldaño Zuñiga, Joseph Chukwudi Okeibunor, Annie Lu Nguyen

**Affiliations:** 1 Mental Health and Wellness Study Group, Ile-Ife, Nigeria; 2 Department of Child Dental Health, Obafemi Awolowo University, Ile-Ife, 22005 Nigeria; 3 Department of Mental Health, Obafemi Awolowo University, Ile-Ife, Nigeria; 4 Department of Pediatric Dentistry and Dental public Health, Faculty of Dentistry, Alexandria University, Alexandria, Egypt; 5 School of Medicine, University of Sierra Sur, Oaxaca, Mexico; 6 Government College for Women, Maulana Azad Road, Srinagar, J&K India; 7 Department of Health Policy Planning and Management, School of Public Health, University of Health and Allied Sciences, Ho, Ghana; 8 Department of Substitutive Dental Sciences, College of Dentistry, Imam Abdulrahman Bin Faisal University, Dammam, Saudi Arabia; 9 Department of Preventive Dental Sciences, College of Dentistry, Imam Abdulrahman bin Faisal University, Dammam, Saudi Arabia; 10 Department of Oral and Maxillofacial Surgery, Faculty of Dentistry, Syrian Private University, Damascus, Syria; 11 Clinical Sciences Department, Nigerian Institute of Medical Research, Lagos, Nigeria; 12 Centre for Rural Health, School of Nursing and Public Health, University of KwaZulu-Natal, Durban, South Africa; 13 Department of Preventive Dental Sciences, Jazan University, Jazan, Saudi Arabia; 14 Department of Health Promotion, Faculty of Health, Medicine and Life Sciences, Maastricht University, Maastricht, The Netherlands; 15 School of Biological Sciences, University of the Punjab, Quaid-e-Azam Campus, Lahore, Pakistan; 16 Department of Periodontology and Community Dentistry, University of Ibadan and University College Hospital, Ibadan, Nigeria; 17 Regent’s University London, London, United Kingdom; 18 Department of Economic and Social Geography, University of Szeged, Szeged, Hungary; 19 Department of Psychology, Miami University Oxford, Ohio, USA; 20 Department of Child Oral Health, University of Ibadan, Ibadan, Nigeria; 21 Department of Preventive Dental Sciences, Division of Dental Public Health, College of Dentistry, Jazan University, Jazan, Kingdom of Saudi Arabia; 22 Department of Maternity & Childhood Nursing, College of Nursing, Taibah University, Madinah, Kingdom of Saudi Arabia; 23 Department of Preventive and Biomedical Science, College of Dentistry, University of Science & Technology, Sanaa, Yemen; 24 Department of Family and Occupational Medicine, Faculty of Medicine, University of Debrecen, Debrecen, Hungary; 25 Doctoral School of Health Sciences, University of Debrecen, Debrecen, Hungary; 26 Institute of Zoology, University of the Punjab, Quaid-i-Azam Campus, Lahore, 54590 Pakistan; 27 Faculty of Medicine, University of Turku, Turku, Finland; 28 Postgraduate Department, University of Sierra Sur, Oaxaca, Mexico; 29 Research Development and Innovations, Assistant Regional Director Cluster, WHO Regional Office for Africa, Brazzaville, Congo; 30 Department of Family Medicine, University of Southern California, Keck School of Medicine, Los Angeles, CA USA

**Keywords:** HIV, PTSD, Nigeria, COVID-19, SARS-COV-2, Mental health disorder

## Abstract

**Background:**

Nigeria is a country with high risk for traumatic incidences, now aggravated by the COVID-19 pandemic. This study aimed to identify differences in COVID-19 related post-traumatic stress symptoms (PTSS) among people living and not living with HIV; to assess whether PTSS were associated with COVID-19 pandemic-related anger, loneliness, social isolation, and social support; and to determine the association between PTSS and use of COVID-19 prevention strategies.

**Methods:**

The data of the 3761 respondents for this analysis was extracted from a cross-sectional online survey that collected information about mental health and wellness from a convenience sample of adults, 18 years and above, in Nigeria from July to December 2020. Information was collected on the study’s dependent variable (PTSS), independent variables (self-reported COVID-19, HIV status, use of COVID-19 prevention strategies, perception of social isolation, access to emotional support, feelings of anger and loneliness), and potential confounder (age, sex at birth, employment status). A binary logistic regression model tested the associations between independent and dependent variables.

**Results:**

Nearly half (47.5%) of the respondents had PTSS. People who had symptoms but were not tested (AOR = 2.20), felt socially isolated (AOR = 1.16), angry (AOR = 2.64), or lonely (AOR = 2.19) had significantly greater odds of reporting PTSS (*p* < 0.001). People living with HIV (AOR = 0.39), those who wore masks (AOR = 0.62) and those who had emotional support (AOR = 0.63), had lower odds of reporting PTSS (*p* < .05).

**Conclusion:**

The present study identified some multifaceted relationships between post-traumatic stress, HIV status, facemask use, anger, loneliness, social isolation, and access to emotional support during this protracted COVID-19 pandemic. These findings have implications for the future health of those affected, particularly for individuals living in Nigeria. Public health education should be incorporated in programs targeting prevention and prompt diagnosis and treatment for post-traumatic stress disorder at the community level.

## Background

Post-traumatic stress disorder (PTSD) is a psychiatric disorder that may occur in people who have experienced or witnessed a psychologically traumatic event such as unexpected death, immediate threat to life, serious accidents and disasters, physical injury to another person, or who have been threatened by a serious event such as serious physical injury or sexual violence [1]. Reactions to these traumatic events include avoidance of reminders of the trauma, problems concentrating, concern for safety, intrusive thoughts, irritability, nightmares, and self-blame [2, 3]. PTSD develops when these reactions persist for a month or more and cause substantial distress and disruption to one’s life [3].

The COVID-19 pandemic is a cause of psychological trauma as people are exposed to unexpected deaths or threats of death [4]. Healthcare workers who have had to manage COVID patients may be witnessing increased illnesses and deaths [5, 6]. Patients admitted to the hospital with COVID-19 experience social isolation, physical discomfort, and fear for survival [7]. In addition, families and friends of patients with COVID-19 have concerns about infection, isolation, and death of loved ones [3]. These experiences increase the risk for developing PTSD especially when the individual lacks immediate social support and feels socially isolated (like when in quarantine or isolation) [8, 9], or may face stigma because of COVID-19 status [10, 11].

One of the strongest predictors of PTSD after exposure to a trauma is previous exposure to trauma. Specifically, people living with HIV (PLHIV) may have previous trauma due to their medical health status and have higher risks for PTSD compared to those not living with HIV [12]. For many, the diagnosis of HIV is itself a traumatic event [13]. Also, many PLHIV live in environments characterized by poverty, violence, and lack of social support [14–16] which are factors associated with higher risk for PTSD [17]. For instance, women living with HIV have a higher rate of sexual assault than women in the general population [12]. PTSD may also result from the social stigma associated with being HIV positive [18], deteriorating quality of life [19], and is the most common mental health challenge faced by PLHIV across the world [20].

A large population of PLHIV in Nigeria experience PTSD due to intense HIV-related stigmatizing events or situations. The rate of HIV-stigma related PTSD in Nigeria is 27.4% [21]. The risk of experiencing HIV-stigma related PTSD is increased by a history of traumatic events and poor access to social support [21] and self-stigma [22]. HIV-related stigma and social isolation is a major barrier to serostatus disclosure with huge implications for access to treatment for PLHIV in Nigeria. On the other hand, access to social support improves health and wellbeing [23]. Access to social support was reported to produce better outcomes for PLHIV in Nigeria than self-efficacy building therapy [24]. For many, social support is received mainly from individuals who aid them at home, including the family, friends, and peers living with HIV who provide emotional, spiritual, and physical care [24]. Access to these social support mechanisms by PLHIV may have been reduced during the COVID-19 lockdown as the national directives for the COVID-19 control included restricted movements, banned travels from countries with ongoing high transmission, locked borders and requiring citizens to compulsory use of face masks, banned social gathering and encouraging regular handwashing [25]. PLHIV who become infected with COVID-19 may also face new stigma and be at increased risk for PTSD.

Among individuals with PTSD, the amygdala processes peritraumatic hyperarousal to produce hyperarousal symptoms such as anger and recklessness [26]. PTSD also has a bidirectional relationship with emotional and social loneliness [27]. The association between PTSD and loneliness may reflect an overlap because people who experience both have a persistent sense of threat and hypervigilance for threats [28, 29], the shared experience of trauma exposure [30], avoidance symptoms [31], and negative evaluation of the world [32]. Social support is protective against the development of PTSD [33] while social isolation influences the predisposition of people to and onset of PTSD [34].

This study examines the relationships between PTSD symptoms, henceforth referred to as PTSS, and pandemic-related emotional and behavioural outcomes. The first aim of this study was to identify differences in reported PTSS between PLHIV and people not living with HIV. The second aim was to assess the association between PTSS and anger, loneliness, social isolation, and social support. The third aim was to determine the association between PTSS and risk for non-compliance with COVID-19 prevention strategies as a manifestation of recklessness symptoms. We hypothesize that more PLHIV compared to people not living with HIV will have PTSS during the COVID-19 pandemic; PTSS will be positively associated with anger, loneliness, social isolation, and poor access to social support during the COVID-19 pandemic; and PTSS will be positively associated with low use of COVID-19 prevention strategies. The study was conducted among adults in Nigeria. Studies on PTSD in Nigeria have been limited to prevalence in different populations [35, 36]. Yet for a country with a high risk for traumatic incidences [37, 38] now coupled with the COVID-19 pandemic, it is important to understand how COVID-19 related factors are associated with PTSS as this may help in the design and implementation of PTSD management.

## Methods

We analysed data from a multi-country, cross-sectional survey about the impact of the COVID-19 pandemic on the mental health and wellness of adults. This current analysis focused on data collected from adults 18 years and above, living in Nigeria in the period from July to December 2020. The study participants were recruited through respondent-driven sampling. Initial participants were asked to share the survey link with their contacts to facilitate recruitment (snowball sampling). The survey link was also posted on social media platforms (Facebook, Twitter, and Instagram, WhatsApp). Ethical approval of the current study was obtained from the Human Research Ethics Committee at the Institute of Public Health of the Obafemi Awolowo University Ile-Ife, Nigeria (HREC No: IPHOAU/12/1557). Prior publications have more details on the study methodology [39–41].

### Study instruments

The questionnaire was preceded by a brief introduction explaining the purpose of the study, and assuring participants of their voluntary participation, and the confidentiality of their data. The questionnaire took an average of 11 min to complete and was administered in English. Each participant could only complete a single questionnaire through IP address restrictions though they could edit their answers freely until they chose to submit.

### Dependent variable

#### Post-traumatic stress symptoms

The PTSD checklist for civilians was used to measure the level of post-traumatic stress symptoms (PTSS) that respondents had. The checklist is a 17-item self-report questionnaire that measured PTSS [42]. It prompted respondents to measure the level of stress that they have pertaining to a problem or complaint in response to a stressful life experience (in this case the COVID-19 pandemic) over the past month. A 5-point scale was used for respondents to rate their responses (1- not at all to 5- extremely). The possible scores ranged from 17 to 85. The cut-off of 28 was used to categorize “no PTSS” (17-27) vs. “PTSS present” (28-85) [43]. Studies have demonstrated good test-retest reliability, internal consistency, and discriminant validities of the PTSD checklist for civilians [44]. In a study comparing its construct validity to that of the Civilian Mississippi Scale, the PTSD checklist for civilians showed greater construct validity [45]. Excellent internal consistency has been demonstrated in other studies [42, 46] and the Cronbach alpha score for the current sample of respondents from Nigeria was 0.949.

### Independent variables

#### COVID-19 status

Respondents were asked if they had tested positive for COVID-19, had COVID-19 symptoms but did not test, had a close friend who tested positive for COVID-19 and/or knew someone who died from COVID-19. The response was either a ‘yes’ or a ‘no’.

#### HIV status

A question was also asked about HIV status. Respondents were required to identify if their HIV status was positive, negative, unknown or if they were unwilling to declare. The respondents who identified as HIV positive were described as “living with HIV”, while the respondents who identified as HIV negative, unknown or unwilling to declare were described as “not living with HIV”.

#### Use of COVID-19 prevention strategies

The questionnaire assessed adoption of COVID-19 prevention strategies (wearing of face masks, frequent washing or sanitizing hands, physical distancing) and workplace modification (working remotely). Respondents were asked which of the listed behaviours they adopted during the pandemic. Respondents could select more than one item to indicate that they adopted multiple behaviours during the pandemic. The questions were included as a component of the Pandemic Stress Index [47].

#### Social isolation

Respondents were asked about their feeling of social isolation on a scale of 1 - not at all - to 10 - extremely. The question was adopted from Nguyen et al. [48].

#### Emotional support

The Pandemic Stress Index is a 3-item measure. One of the measures assessed was emotional support from friends and family. A check on this item indicated access to support [47].

#### Anger and loneliness

Respondents indicated the feelings they were experiencing during COVID-19 on an emotions checklist. Two of the eight emotions were anger and loneliness. The questions were part of the Pandemic Stress Index administered to study participants that assessed the psychosocial impact of COVID-19 [47].

### Confounders

#### Sociodemographic profile

The section on sociodemographic profile collected data on age, sex at birth (male, female, intersex, decline to answer), highest level of education attained (none, primary, secondary, college/university), and employment status (unemployed, employed, student, retired).

### Statistical analysis

Raw data were downloaded as SPSS® file Version 23.0 (IBM Corp. Armonk, NY, USA). We identified and removed survey responses completed below 7 min – the lower limit of the time range to answer the questionnaire during the pilot phase conducted with 40 participants not included in the final study data (*n* = 77). The pilot was part of the construct validation process conducted for the study questionnaire, which verified that the content validity index was 0.82. Those with incomplete data to responses on the PTSD checklist were also excluded from the study (*n* = 678) [49, 50].

Descriptive analysis of all study variables was conducted. T-test and chi square test were used to assess the relationship between the dependent variable, independent variables, and confounders. A binary logistic regression model was constructed to identify explanatory factors associated with PTSS after adjusting for potential confounders. Adjusted odds ratios (AOR) and 95% confidence intervals (CIs) were calculated. The Omnibus test of model coefficients was used to determine the significant difference between the Log- (−2LLs) of the baseline model and the new model inclusive of the explanatory variable. Statistical significance was set at 5%.

## Results

Table 1 shows that there were 3761 study participants with a mean (standard deviation) age of 38.3 (11.5) years. Of the 3761 study participants, 1787 (47.5%) had PTSS based on the cut-off score. Most respondents were female (52.7%), had tertiary education (81.0%), and were employed (71.2%). Only 2.5% tested positive for COVID-19, 10.6% had COVID-19 symptoms but did not get tested, 16.9% had a close friend who tested positive, and 31.7% knew someone who died of COVID-19. Also, 796 (21.2%) respondents were PLHIV, 3165 (84.2%) wore face masks, 3106 (82.6%) frequently washed or sanitized their hands, 2885 (76.7%) kept physical distance, and 1246 (33.1%) worked remotely. In addition, 289 (7.7%) respondents reported no access to emotional support, 239 (6.4%) felt angry about the pandemic, and 579 (15.4%) experienced loneliness.

**Table 1:** Profile of respondents and associations with post traumatic stress symptoms among respondents *N* = 3761

Variables	Total *N* = 3761 n (%)	PTSS		*P* value
		Yes 1787 n (%)	No 1974 n (%)	
**Sex at birth**				
Male	1759 (46.8)	780 (44.3)	979 (55.7)	**0.002**
Female	1983 (52.7)	997 (50.3)	986 (49.7)	
Intersex	2 (0.1)	2 (100.0)	0 (0.0)	
Decline to answer	17 (0.5)	8 (47.1)	9 (52.9)	
**Highest educational level**				
No formal education	45 (1.2)	40 (88.9)	5 (11.1)	**< 0.001**
Primary	74 (2.0)	51 (68.9)	23 (31.1)	
Secondary	594 (15.8)	368 (62.0))	226 (38.0)	
Tertiary	3048 (81.0)	1328 (43.6)	1720 (56.4)	
**Employment status**				
Unemployed	568 (15.1)	373 (65.7)	195 (34.3)	**< 0.001**
Employed	2677 (71.2)	1138 (42.5)	1539 (57.5)	
Student	423 (11.2)	239 (56.5)	184 (43.5)	
Retired	93 (2.5)	37 (39.8)	56 (60.2)	
**COVID-19 status**				
*Tested positive for COVID-19*				
No	3667 (97.5)	1749 (47.7)	1918 (52.3)	0.163
Yes	94 (2.5)	38 (40.4)	56 (59.6)	
*Had symptoms but no test*				
No	3364 (89.4)	1526 (45.4)	1838 (54.6)	**< 0.001**
Yes	397 (10.6)	261 (65.7)	136 (34.3)	
*Had a close friend who tested positive*				
No	3126 (83.1)	1520 (48.6)	1606 (51.4)	**0.002**
Yes	635 (16.9)	267 (42.0)	368 (58.0)	
*Knew someone who died of COVID-19*				
No	2569 (68.3)	1265 (49.2)	1304 (50.8)	**0.002**
Yes	1192 (31.7)	522 (43.8)	670 (56.2)	
**HIV status**				
Not living with HIV	2965 (78.8)	1245 (42.0)	1720 (58.0)	**< 0.001**
Living with HIV	796 (21.2)	542 (68.1)	254 (31.9)	
**COVID-19 preventive measures**				
*Wearing face masks*				
No	596 (15.8)	343 (57.6)	253 (42.4)	**< 0.001**
Yes	3165 (84.2)	1444 (45.6)	1721 (54.4)	
*Frequent hand washing and sanitising*				
No	655 (17.4)	349 (53.3)	306 (46.7)	**0.001**
Yes	3106 (82.6)	1438 (46.3)	1668 (53.7)	
*Physical distancing*				
No	876 (23.3)	469 (53.5)	407 (46.5)	**< 0.001**
Yes	2885 (76.7)	1318 (45.7)	1567 (54.3)	
*Working remotely*				
No	2515 (66.9)	1241 (49.3)	1274 (50.7)	**0.001**
Yes	1246 (33.1)	546 (43.8)	700 (56.2)	
**Social isolation**				
Mean (SD)	5.1 (2.6)	5.7 (2.4)	4.6 (2.6)	**< 0.001**
**Access to emotional support**				
No	289 (7.7)	212 (73.4)	77 (26.6)	**< 0.001**
Yes	3472 (92.3)	1575 (45.4)	1897 (54.6)	
**Anger**				
No	3522 (93.6)	1596 (45.3)	1926 (54.7)	**< 0.001**
Yes	239 (6.4)	191 (79.9)	48 (20.1)	
**Loneliness**				
No	3182 (84.6)	1378 (43.3)	1804 (56.7)	**< 0.001**
Yes	579 (15.4)	409 (70.6)	170 (29.4)	

Compared to people without PTSS, people with PTSS were significantly more likely to be younger (36.7 (11.2) years vs 39.8 (11.6) years; *p* < 0.001), female, have no formal education, and unemployed. People who experienced symptoms of COVID-19 without being tested, had a close friend who had not tested positive for COVID-19, or did not know someone who died of COVID-19 were also significantly more likely to have PTSS. People who reported not wearing face masks, not washing or sanitising hands frequently, not practicing physical distancing, and not working remotely from home were significantly more likely to have PTSS. Further, those who felt socially isolated, had no emotional support, and those who felt angry or lonely were more significantly more likely to have PTSS.

Results from the regression model are shown in Table 2. Feeling socially isolated (AOR: 1.16; 95% CI: 1.12-1.19; *p* < 0.001), angry (AOR: 2.64; 95% CI: 1.86-3.76; *p* < 0.001), lonely (AOR: 2.19; 95% CI: 1.77-2.70; *p* < 0.001), and having symptoms of COVID-19 but not getting tested (AOR: 2.20; 95% CI: 1.72-2.80; *p* < 0.001) were significantly associated with higher odds of reporting PTSS. The only COVID-19 preventive measure associated with PTSS was wearing facemasks, where respondents who reported wearing face masks had significantly lower odds of reporting PTSS (AOR: 0.62; 95% CI: 0.50-0.78; *p* < 0.001).

**Table 2:** Logistic regressions analysis of the factors associated with post traumatic stress symptoms (*N* = 3761)

Variables	AOR (95% CI)	*p* value
**Age in years**	0.98 (0.97-0.99)	**< 0.001**
**Sex at birth**		
Male (ref: non-male)	0.85 (0.74-0.98)	**0.029**
**Highest educational level**		
No formal education	1.00	
Primary	0.30 (0.10-0.92)	**0.035**
Secondary	0.29 (0.11-0.78)	**0.015**
Tertiary	0.26 (0.10-0.71)	**0.008**
**Employment status**		
Employed	0.73 (0.62-0.87)	**< 0.001**
**COVID-19 status**		
*Tested positive for COVID-19*		
Yes (ref: no)	0.69 (0.43-1.11)	0.124
*Had symptoms but no test*		
Yes (ref: no)	2.20 (1.72-2.80)	**< 0.001**
*Had a close friend who tested positive*		
Yes (ref: no)	0.93 (0.76-1.15)	0.513
*Knew someone who died of COVID-19*		
Yes (ref: no)	1.10 (0.93-1.29)	0.270
**HIV status**		
Living with HIV	0.39 (0.31-0.47)	**< 0.001**
**COVID-19 preventive measures**		
*Wearing face masks*		
Yes (ref: no)	0.62 (0.50-0.78)	**< 0.001**
*Hand washing*		
Yes (ref: no)	0.95 (0.76-1.19)	0.652
*Physical distancing*		
Yes (ref: no)	1.10 (0.92-1.31)	0.308
*Working remotely*		
Yes (ref: no)	1.08 (0.92-1.27)	0.332
**Social Isolation**	1.16 (1.12-1.19)	**< 0.001**
**Access to emotional support**		
Yes (ref: no)	0.63 (0.46-0.85)	**0.002**
**Anger**		
Yes (ref: no)	2.64 (1.86-3.76)	**< 0.001**
**Loneliness**		
Yes (ref: no)	2.19 (1.77-2.70)	**< 0.001**
Nagelkerke R^2^	0.217	
Omnibus test of model coefficients	668.58	**< 0.001**

Living with HIV (AOR: 0.39; 95% CI: 0.31-0.47; *p* < 0.001), having access to emotional support (AOR: 0.63; 95% CI: 0.46-0.85; *p* = 0.002), being younger (AOR: 098; 95% CI: 0.97-0.99. *p* < 0.001), being male (AOR: 0.85; 95% CI: 0.74-0.98; *p* = 0.029), having primary, secondary or tertiary education (*p* < 0.05) and being employed (AOR: 0.73; 95% CI: 0.62-0.87; *p* < 0.001) were significantly associated with lower odds of reporting PTSS.

## Discussion

In this study, PTSS was found to be associated with feeling angry, lonely, and socially isolated during the COVID-19 pandemic among adults in Nigeria. The only COVID-19 prevention strategy associated with PTSS was wearing facemasks with those who wore face masks having lower odds of PTSS. People who had access to emotional support and PLHIV also had lower odds of PTSS. The study hypotheses were therefore only partially supported.

This study is one of the few providing empirical evidence on the factors associated with PTSS during the COVID-19 pandemic and the first to identify an association in an African population. It is also the first to discuss PTSS among PLHIV during the COVID-19 pandemic in a region of the world with the highest prevalence and burden of HIV [51]. The large sample size and small confidence intervals support the precision of the study estimates. However, one of the limitations of this study is that it used a convenient sample with greater representation of persons with tertiary education. The study outcome may therefore be less generalizable to people with lower or non-formal education in Nigeria. This is also a cross-sectional study and so we are unable to conclude on a causal relationship between PTSS and the explanatory variables studied. Exposure to potentially traumatic events such as violence, assault, abuse, or combat were not measured in this study, thus other factors may contribute to the PTSS variance in the sample. Nevertheless, the study findings are consistent with some prior study findings which may support the validity of results to plan interventions for Nigerians during the ongoing COVID-19 pandemic.

The associations found between PTSS and anger, loneliness, and social isolation during the COVID-19 pandemic are supported by prior studies conducted before [52–54] and during [9, 55] the pandemic that show similar associations. The protracted duration of the COVID-19 pandemic has implications for the persistence of these associations over time. Sensitization and kindling may cause the progressive escalation of PTSS, and with time, may lead to the onset of PTSD [56]. Repeated environmental triggering of traumatic memories increases the dysregulation of individuals’ neurobiology resulting in chronic musculoskeletal pain, hypertension, hyperlipidaemia, obesity, and cardiovascular disease [57]. It is therefore important for governments to put in place public health outreach and support mechanisms for people who feel anger, loneliness, and social isolation during the COVID-19 pandemic [57]. Because the Nigerian government is largely dependent on donor support for its health response, Nigeria’s COVID-19 response basket fund and other sources of donor funding [58] may best be utilised to address potential gaps in providing mental health support for those in need of this support during a pandemic.

Furthermore, we found that having access to emotional support during the COVID-19 pandemic was associated with lower odds of PTSS. This is similar to prior findings on the association between PTSD and disastrous events [59] as well as PTSD and the COVID-19 pandemic [60]. Emotional support enhances resilience [61], while pre-event emotional support creates a buffer-effect and mitigates against post-event PTSD [62, 63].

According to our study findings, PTSS may be associated with low use of face mask. As of June 2021, less than 5% of the population in Nigeria was vaccinated against COVID-19 infection [64]. Thus, the country may face multiple cycles of the pandemic because of the large proportion of persons not using face masks in addition to low vaccination rates. This is an important reason for health policy planners in Nigeria to urgently address the mental health needs of people with PTSS to prevent new waves of COVID-19 infection. There is also a need for public health education to use facemasks in addition to programs to prevent, diagnose and treat PTSD at community level since the utilisation of health care services is poor in Nigeria [65–67].

Being male, having a level of formal education, and having employment were associated with lower odds of having PTSS. Some studies indicate that PTSD is more common in females because they have a more sensitized hypothalamus–pituitary–axis than males [68]. Other sex and gender differences in brain function and behaviour also elevate the risk for PTSD in women compared to men. Women report experiencing more high-impact trauma at a younger age compared to men and have an associated increase in need for social support [68]. The finding is in line with a study on the incidence of PTSD in Italy where women displayed higher level of anxiety during the pandemic [63]. Also, higher education status improves employment opportunity and socio-economic status thereby enabling individuals to gain access to social and economic resources to cope with pandemic related stress [69]. In Nigeria, females are more economically and socially disadvantaged than men [70], which further increases their predisposition to PTSD. Prior research indicated that low educational status can be a significant risk indicator for PTSD in adults [71]. This combination of predisposing factors for higher prevalence of PTSD in females during the COVID-19 pandemic is an indication of the need for a gender sensitive COVID-19 response for adults in Nigeria.

Our findings show that being younger was associated with higher odds of having PTSS. Younger people may experience more PTSD during disasters [63, 71] because older age promotes more adaptive coping styles [63, 72]. Also, younger individuals are more vulnerable to different dimensions of psychological distress than older generations, are least prepared to face trauma, or may be more informed about the nature of Coronavirus than their older counterparts [63]. The effect of disasters may, however, be more dependent on the social, economic, cultural, and historical context of the disaster-stricken setting than with age [72] even during the COVID-19 pandemic [73] and thus, findings on age associated PTSS during the pandemic may possibly vary between cultures and countries. Also, age-associated PTSD has been found for females and not for males [74] thereby increasing the possibility for a non-linear relationship between age and PTSS.

Study findings indicated that respondents who had symptoms of COVID-19, but were not tested, had higher odds of having PTSS. The relationship between the two variables may be bidirectional, as not testing may be a result of PTSS while PTSS may also result in people with symptoms who do not test. People who had symptoms of COVID-19, but did not test may be concerned with the consequences of confirming COVID-19 infection. Fear, anxiety, anger, depression, and guilt are common reactions to COVID-19 infection as well as trauma. Although most people exposed to trauma do not develop long-term PTSD, getting timely help and support may prevent stress reactions from getting worse and developing into PTSD. Possible sources of support such as peers, family, and the faith community may be empowered so that they are available to prevent people turning to unhealthy coping methods such as misuse of alcohol or drugs [75]. The low numbers of confirmed COVID-19 cases compared to those suspected of having COVID-19 but not tested points to the low level of testing in Nigeria like many low- and middle-income countries [25]. Understanding the association reported here may help with the development of policies and guidelines on COVID-19 testing that may help improve the COVID-19 testing in Nigeria and other low- and middle-income countries.

Incongruously, living with HIV was associated with lower odds of having PTSS. HIV and COVID-19 intersectional studies had indicated the increased risk of PTSD for PLHIV who have experienced traumatic events like intimate partner violence, isolation, discrimination, stigma, and challenges with obtaining treatment [76]. Although we did not explore the history of traumatic events for respondents living with HIV in this study, past studies have indicated that PLHIV are at high risk of facing these traumatic events [77, 78]. However, it is also possible that years of experience handling traumatic events may have helped PLHIV develop a positive sense of self, relationship, philosophy of life, and trauma coping strategies for COVID-19 related trauma [79]. PLHIV may therefore be more resilient to the shocks associated with the pandemic and resilience can reduce the risk for PTSD [80].

This study provides evidence suggestive of the need for a policy thrust that recognises the mental health and emotion related complications that may have resulted from the COVID-19 directives, and the need to put in place supportive systems and structures to address these complications. Policies need to be based on empathy. A whole-of-society approach need to be instituted to promote, protect, and care for mental health in addition to ensuring widespread availability of emergency mental health and psychosocial support mechanisms and systems through collaborative efforts with civil society organisations; many of whom remain the first responders in the time of crisis [81]. The current national responses did not include programs for access to mental health services. In the future, it is important to plan for citizens’ recovery from health crisis like that witnessed during the COVID-19 pandemic by building and ensuring access to mental health services.

## Conclusion

The high prevalence of PTSS, low level of wearing facemask by persons with PTSS, and positive associations between PTSS and HIV status, anger, loneliness, social isolation, and access to emotional support during this protracted COVID-19 pandemic has implications for the future health of affected persons. This is particularly salient for individuals living in Nigeria, where public health education and support for the use of facemask should be incorporated into programs targeting the prevention, diagnosis, and treatment for PTSD and PTSS at the community level.

## Data Availability

All data generated for this study are presented in the manuscript. Patient-level data can however be accessible on reasonable request from one of the authors, Morenike Oluwatoyin Folayan.

## Abbreviations

AOR: Adjusted odds ratio
CI: Confidence interval
COVID-19: Coronavirus 2019
HIV: Human immunodeficiency virus
PLHIV: People living with HIV
PTSD: Post traumatic stress disorder
PTSS: Post traumatic stress symptoms
