# Correction to: Factors associated with COVID-19 pandemic induced post-traumatic stress symptoms among adults living with and without HIV in Nigeria: a cross-sectional study

**DOI:** 10.1186/s12888-022-03751-3

**Published:** 2022-02-23

**Authors:** Morenike Oluwatoyin Folayan, Olanrewaju Ibigbami, Maha ElTantawi, Giuliana Florencia Abeldaño, Eshrat Ara, Martin Amogre Ayanore, Passent Ellakany, Balgis Gaffar, Nuraldeen Maher Al-Khanati, Ifeoma Idigbe, Anthonia Omotola Ishabiyi, Mohammed Jafer, Abeedah Tu-Allah Khan, Zumama Khalid, Folake Barakat Lawal, Joanne Lusher, Ntombifuthi P. Nzimande, Bamidele Emmanuel Osamika, Bamidele Olubukola Popoola, Mir Faeq Ali Quadri, Mark Roque, Anas Shamala, Ala’a B. Al-Tammemi, Muhammad Abrar Yousaf, Jorma I. Virtanen, Roberto Ariel Abeldaño Zuñiga, Joseph Chukwudi Okeibunor, Annie Lu Nguyen

**Affiliations:** 1 Mental Health and Wellness Study Group, Ile-Ife, Nigeria; 2 Department of Child Dental Health, Obafemi Awolowo University, Ile-Ife, 22005 Nigeria; 3 Department of Mental Health, Obafemi Awolowo University, Ile-Ife, Nigeria; 4 Department of Pediatric Dentistry and Dental public Health, Faculty of Den- tistry, Alexandria University, Alexandria, Egypt; 5 School of Medicine, University of Sierra Sur, Oaxaca, Mexico; 6 Government College for Women, Maulana Azad Road, Srinagar, J&K India; 7 Department of Psychology, Miami University Oxford, Ohio, USA; 8 Department of Health Policy Planning and Management, School of Public Health, University of Health and Allied Sciences, Ho, Ghana; 9 Department of Substitutive Dental Sciences, College of Dentistry, Imam Abdulrahman Bin Faisal University, Dammam, Saudi Arabia; 10 Department of Preventive Dental Sciences, College of Dentistry, Imam Abdulrahman bin Faisal University, Dammam, Saudi Arabia; 11 Department of Oral and Maxil- lofacial Surgery, Faculty of Dentistry, Syrian Private University, Damascus, Syria; 12 Clinical Sciences Department, Nigerian Institute of Medical Research, Lagos, Nigeria; 13 Centre for Rural Health, School of Nursing and Public Health, University of KwaZulu-Natal, Durban, South Africa; 14 Department of Preven- tive Dental Sciences, Jazan University, Jazan, Saudi Arabia; 15 Department of Health Promotion, Faculty of Health, Medicine and Life Sciences, Maastricht University, Maastricht, The Netherlands; 16 School of Biological Sciences, Uni- versity of the Punjab, Quaid-e-Azam Campus, Lahore, Pakistan; 17 Department of Periodontology and Community Dentistry, University of Ibadan and Univer- sity College Hospital, Ibadan, Nigeria; 18 Regent’s University London, London, UK; 19 Department of Economic and Social Geography, University of Szeged, Szeged, Hungary; 20 Department of Child Oral Health, University of Ibadan, Ibadan, Nigeria; 21 Department of Preventive Dental Sciences, Division of Dental Public Health, College of Dentistry, Jazan University, Jazan, Kingdom of Saudi Arabia; 22 Department of Maternity & Childhood Nursing, College of Nursing, Taibah University, Madinah, Kingdom of Saudi Arabia; 23 Department of Preventive and Biomedical Science, College of Dentistry, University of Science & Technology, Sanaa, Yemen; 24 Department of Family and Occupational Medicine, Faculty of Medicine, University of Debrecen, Debrecen, Hungary; 25 Doctoral School of Health Sciences, University of Debre- cen, Debrecen, Hungary; 26 Institute of Zoology, University of the Punjab, Quaid-i-Azam Campus, Lahore, 54590 Pakistan; 27 Faculty of Medicine, Univer- sity of Turku, Turku, Finland; 28 Postgraduate Department, University of Sierra Sur, Oaxaca, Mexico; 29 Research Development and Innovations, Assistant Regional Director Cluster, WHO Regional Ofce for Africa, Brazzaville, Congo; 30 Department of Family Medicine, University of Southern California, Keck School of Medicine, Los Angeles, CA USA

**Correction to: BMC Psychiatry 22, 48 (2022)**

**https://doi.org/10.1186/s12888-021-03617-0**

Following the publication of the original article [1], the authors identified that affiliation 7 is incorrect assigned to Eshrat Ara. The correct affiliations are given below.

1 Mental Health and Wellness Study Group, Ile-Ife, Nigeria.

6 Government College for Women, Maulana Azad Road, Srinagar, J&K, India.

The original article [1] has been corrected.

## References

[1] Folayan MO (2022). Factors associated with COVID-19 pandemic induced post-traumatic stress symptoms among adults living with and without HIV in Nigeria: a cross-sectional study. BMC Psychiatry..

